# Validation of a multisubstance online Timeline Followback assessment

**DOI:** 10.1002/brb3.1486

**Published:** 2019-12-02

**Authors:** Renée Martin‐Willett, Timothy Helmuth, Median Abraha, Angela D. Bryan, Leah Hitchcock, Kaitlyn Lee, L. Cinnamon Bidwell

**Affiliations:** ^1^ The University of Colorado Boulder Boulder Colorado

**Keywords:** alcohol, cannabis, online assessment, substance use, Timeline Followback

## Abstract

**Objectives:**

The Timeline Followback (TLFB) was originally developed to assess alcohol consumption patterns (*American Journal of Public Health*, **86**, 1996, 966) and has been increasingly modified for Web‐based use. Additionally, new modes of substance use administration have emerged, creating a need for an adaptable TLFB tool than can capture data such as cannabis product potency or prescription drug use. Our goal was to validate an online TLFB that reliably assesses a wide range of substances in greater detail.

**Methods:**

Using a within‐subjects counterbalanced design, daily substance use data were collected from 50 college students over a 14‐day retrospective period using both the traditional in‐person TLFB and online TLFB (O‐TLFB).

**Results:**

All substance use variables, including detailed measures of cannabis metrics, correlated significantly (*r*'s ranged from .653 to .944, *p* < .001) between TLFB versions. Further, results demonstrated that both the online TLFB and in‐person TLFB demonstrated concurrent validity with both the Alcohol Use Disorders Identification Test (AUDIT) and Marijuana Dependence Scale (MDS).

**Conclusion:**

Overall, the data suggest that this new O‐TLFB demonstrates strong reliability and delivers a versatile and secure tool for substance use assessment that is relevant to a variety of biomedical and psychological research contexts.

## Graphical abstract

Using a within‐subjects counterbalanced design, a new online Timeline Followback (TLFB) tool was validated against the in‐person TLFB and other measures of substance use. Substance use variables, including detailed measures of cannabis metrics, correlated significantly between TLFB versions, suggesting the new online TLFB is an accessible, versatile, and secure tool for substance use assessment that is relevant to a variety of biomedical and psychological research contexts.

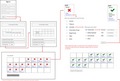

## 1 INTRODUCTION

The Timeline Followback (TLFB) is a thoroughly validated research tool that was developed in 1996 by Sobell et al in order to assess alcohol consumption patterns over discreet time frames (Sobell, Cunningham, & Sobell, 1996). Traditionally, this assessment is in the form of a structured in‐person interview with a research professional. It utilizes a blank calendar in which the participant populates with events unique to their life over a particular period of time (e.g., 14 days). This retrospective tool acts as a visual trigger for reflecting on use patterns, frequency, and quantity for particular substances (e.g., alcohol or tobacco; Beck, Steer, Ball, & Ranieri, 1996; Brown et al., 1998; Pedersen, Grow, Duncan, Neighbors, & Larimer, 2012). Since its development, the TLFB has proven to be a reliable tool among comparable and validated assessments in assessing substance use frequency and quantity patterns (Carey, 2002) in different research contexts (Harris, Golbeck, et al., 2009; Hoeppner, Stout, Jackson, & Barnett, 2010; Robinson, Sobell, Sobell, & Leo, 2014) and in diverse populations including adolescents (Phan et al., 2011), older adults (Aalto, Alho, Halme, & Seppä, 2011), parents (Magee et al., 2014), and psychiatric and medical patients (Carey, Carey, Maisto, & Henson, 2004; Ouimette, Read, Wade, & Tirone, 2010).

As technology continues to advance, the TLFB is increasingly modified for use as a Web‐based assessment (Pedersen et al., 2012; Rueger, Trela, Palmeri, & King, 2012; Wilks et al., 2018). There are numerous benefits to this format, including increased accessibility for participants, which potentially supports retention in longitudinal designs, alleviating the burden of participant travel to research offices and allowing participants to complete follow‐up assessments from any geographic location (Rueger et al., 2012). Online assessments also reduce data collection time and are thought to yield more accurate data on substance use because of their anonymity (Fatseas, Serre, Swendsen, & Auriacombe, 2018; McNeely et al., 2014; Pedersen et al., 2012).

Increasing the versatility of the TLFB beyond the assessment of alcohol and cigarette use could make it a more desirable tool in future research (Carey et al., 2004; Fals‐Stewart, O'Farrell, Freitas, McFarlin, & Rutigliano, 2000; Norberg, Mackenzie, & Copeland, 2012; Panza, Weinstock, Ash, & Pescatello, 2012; Rizzo et al., 2017; Robinson et al., 2014; Weinstock, Whelan, & Meyers, 2004). For example, cannabis is increasingly available and administered through a variety of methods (e.g., flower, concentrates, edibles, or tinctures), and cannabis legalization has led to increasingly diverse and more potent forms of cannabis (Vergara et al., 2017) with direct implications for cannabis‐related public health outcomes (Bidwell, YorkWilliams, Mueller, Bryan, & Hutchison, 2018). Higher proportions of tetrahydrocannabinol (THC) have been associated with greater harm (Volkow, Baler, Compton, & Weiss, 2014), whereas higher levels of cannabidiol (CBD) may be associated with mitigating harm (Bidwell et al., 2018), and exposure to these different cannabinoids and their varying potencies may be hard to characterize through the use of common quantity and frequency measures (Prince, Conner, & Pearson, 2018).

Existing online TLFB assessments have some limitations. First, the user interfaces may not follow the most up‐to‐date best practice guidelines in Web‐based accessibility (Caldwell, Cooper, Loretta Guarino Reid, & Chisholm, 2006), potentially compromising the integrity of data collected by self‐report (e.g., a participant may make an erroneous entry into a suboptimal interface due to sight impairments). Second, few existing tools measure multiple substances such as diverse forms of cannabis, prescription medications, or illicit drugs (Staines, Magura, Foote, Deluca, & Kosanke, 2008). Finally, there is unrealized potential for a Web‐based TLFB user interface to be synchronized with widely used data capture tools such as Research Electronic Data Capture (REDCap; Harris, Taylor, et al., 2009), eliminating the need for secondary data entry by research staff and increasing the security of the health‐related data by utilizing flow automation strategies increasingly practiced in the biomedical and behavioral sciences (Dunn, Cobb, Levey, & Gutman, 2016; Yamamoto, Ota, Akiya, & Shintani, 2017). As such, the goal of this study was to determine the validity of a new online TLFB (O‐TLFB) that is (a) optimized for usability, (b) measures diverse substance use in greater detail, and (c) is synchronized with REDCap in comparison with the interviewer‐administered (i.e., in‐person) version by examining correlations and mean differences between the two modalities. We further sought to test the concurrent validity of the O‐TLFB with other existing standardized measures of alcohol and marijuana use.

## 2 METHODS

### 2.1 Participants and study criteria

Study participants were students attending the University of Colorado Boulder and were drawn from the introductory psychology class credit subject pool. Students have the option to either write a research paper or participate in research studies for course credit. For those who opt to be a research participant, the online subject pool system displays available studies for which the students are eligible and allows interested students to schedule their participation with a researcher at the student's convenience. Interested participants were deemed eligible to participate if they met the following inclusion criteria: (a) aged 18–25; (b) students at the University of Colorado Boulder enrolled in an introductory psychology class credit subject pool; and (c) endorsed using both alcohol *and* cannabis within 7 days of the phone screening. All procedures were reviewed and approved by the institutional review board for the protection of human subjects in research.

### 2.2 Study design

First, this validation study compared frequency measures of substance use between two modalities of the TLFB: the in‐person interview and our online, self‐administered version using a within‐subjects design. Substance use data were collected over a 14‐day retrospective period from each study participant with each TLFB modality, with order of presentation counterbalanced across participants. Second, in order to assess concurrent validity of the online TLFB data and previously validated measures of substance use severity, participants also completed the Alcohol Use Disorders Identification Test (AUDIT) and Marijuana Dependence Scale (MDS), both described in detail below. Participants were informed that they were participating in a study that sought “to learn more about substance use among young adults using different forms of assessments,” but were not informed that a portion of the two assessments would be identical in content, or of the aim of validating the O‐TLFB, in an effort to minimize expectancy effects. As in similar validation studies, all eligible participants subsequently completed both the in‐person and online TLFB modalities within a 3‐day window and were randomly assigned to complete either the online version first or the in‐person version first. Research assistants leading the study were trained by established researchers in the administration of all measures with the supervision of a licensed clinical psychologist.

### 2.3 Measures

#### 2.3.1 Alcohol Use Disorders Identification Test

The AUDIT is a clinical screening instrument developed by the World Health Organization (WHO) to assess categories of risk from problematic drinking to indication of clinical dependence (Babor, Higgins‐Biddle, Saunders, & Monteiro, 2001). The AUDIT is comprised of 10 questions addressing alcohol frequency and dependency, as well as problems caused by alcohol consumption, ultimately distinguishing light drinkers from heavy drinkers (Babor et al., 2001). Since its development, the AUDIT has been a clinically oriented assessment of the severity of alcohol use patterns in many different populations, including college students (DeMartini & Carey, 2012). The format of the AUDIT does not instruct respondents to reflect on a specific retrospective period of time for five of ten items, while the remaining items instruct respondents to reflect on the previous year. In the current study, the AUDIT was utilized to assess concurrent validity by observing whether the severity of alcohol use reported on the AUDIT corresponded to usage patterns observed with the in‐person and online TLFBs. The AUDIT was administered by a research team member at the in‐person assessment.

#### 2.3.2 Marijuana Dependence Scale

The MDS is an 11‐item self‐report assessment of dependence severity (Stephens, Roffman, & Curtin, 2000). The MDS is widely utilized in studies that assess marijuana dependency in a variety of populations such as adolescents and adults, proving to be a reliable measure (Callahan, Caldwell Hooper, Thayer, Magnan, & Bryan, 2013; Hendershot, Magnan, & Bryan, 2011; Lozano, Stephens, & Roffman, 2006). In this study, the MDS was utilized to assess concurrent validity by observing whether the severity of marijuana dependence reported on the MDS corresponded to usage patterns observed with the in‐person and online TLFBs. For the MDS, participants were asked to reflect on the previous 14 days of use. The MDS and AUDIT were both administered to participants with the assistance of research assistants and preceding the in‐person TLFB interview.

#### 2.3.3 In‐person Timeline Followback and online Timeline Followback

The O‐TLFB was developed to capture substance use data while being optimized for Web accessibility that would replicate the benefits of an in‐person structured interview as closely as possible. The approach that was taken to development of the tool and the technical details of development are described elsewhere (Martin‐Willett et al., 2019) The current O‐TLFB can be found at http://cuchangeotlfb.org.

Participants completed an O‐TLFB that queried their use of alcohol, nicotine/tobacco, cannabis, prescription drugs, and illicit drugs over a 14‐day retrospective time frame. The 14‐day time frame was selected because the study teams in our center routinely use a 14‐day in‐person TLFB in the context of existing and future protocols. Importantly, however, previous research has demonstrated that correlations between 14‐ and 30‐day TLFBs were high (Fiellin, McGinnis, Maisto, Justice, & Bryant, 2013) and that shorter recall windows may be more accurate for assessments involving fine‐grained detail (Hoeppner et al., 2010). It should also be noted that the online tool was flexibly designed to allow for data collection for any number of retrospective days, for example, 10, 14, 30, and 60 days.

**Figure 1:**
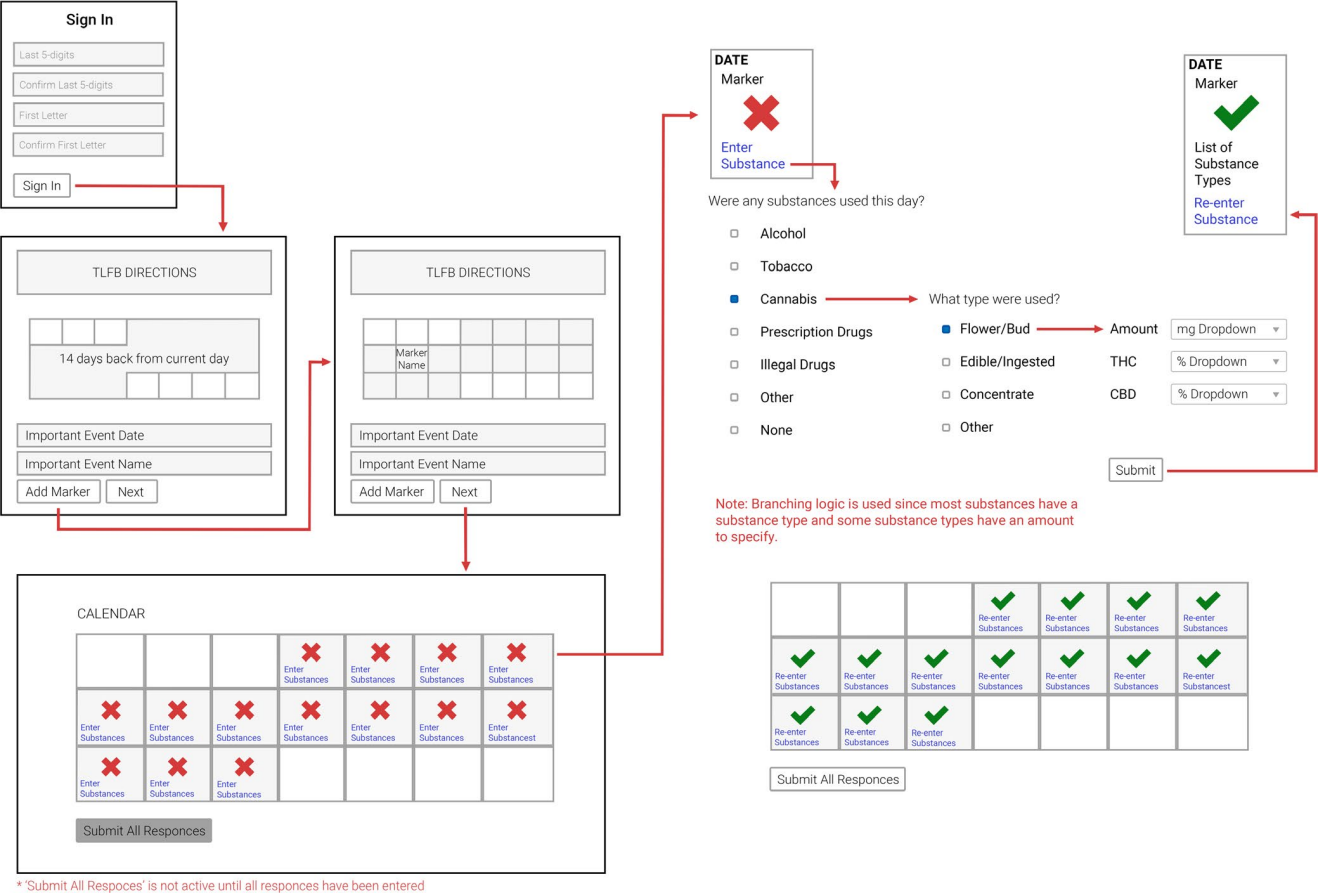
Visualization of the online Timeline Followback interface

First, the participant utilized an interactive calendar to add marker dates for events that would serve as recall aids (e.g., travel or a recent celebration) day‐by‐day for the previous 14 days. They were then prompted to endorse whether they had consumed any substances falling within the broad categories of nicotine/tobacco, alcohol, cannabis, prescription medications, and illicit drugs for each day (an exhaustive list of the substances queried in the context of the O‐TLFB interface can be viewed in Appendix S1). Finally, participants were asked to provide granular details in terms of the route of administration, potency, and form of their substance use for every broad category that was endorsed for each day. For example, if a participant endorsed using “nicotine/tobacco” on a given day, they then had the opportunity not just to report cigarette smoking, but also e‐cigarettes, chew/dip, cigars, hookah, or other forms of nicotine or tobacco use and then provide corresponding quantity information (see Figure 1 for a visualization of the O‐TLFB interface).

The in‐person TLFB was the final assessment completed during the in‐person visit following demographics, the MDS, and AUDIT, and queried the exact same substance use content as the O‐TLFB. Consistent with recommendations and standard practice for administering the in‐person TLFB, the study visit was conducted in a private room and the interviewer utilized a paper retrospective calendar and paper entry forms. Consistent with the O‐TLFB and prior to reporting on their substance use, participants were asked to indicate marker dates on the paper calendar which they utilized to assist with recall for substance use on particular days.

### 2.4 Data analysis

To assess the reliability of the O‐TLFB, the team employed correlational analyses and paired sample *t* tests to examine the associations between responses to the in‐person versus the online TLFB. Consistent with prior work validating substance use consumption assessments (Pedersen et al., 2012; Rueger et al., 2012), primary measures consisted of days that cannabis, alcohol, nicotine/tobacco, illicit drugs, and prescription drugs were used over the prior 14 days. In order to assess the validity of our detailed quantity and frequency measures, secondary measures consisted of days of use across the distinct forms of cannabis (e.g., smoked flower, edible, and concentrated cannabis), as well as amount (and potency where relevant) of specific forms of cannabis, alcohol, and nicotine/tobacco consumed per occasion. Concurrent validity was assessed by comparing the alcohol and marijuana use patterns on the online and in‐person TLFB assessments to the total scores from the AUDIT and MDS. This analytical approach has precedent in previous validation studies of online TLFB assessments (Rueger et al., 2012). Additionally, ancillary measures such as the THC and CBD potency of the different forms of cannabis and the use of marker dates during the O‐TLFB were evaluated post hoc to further characterize cannabis use and substance use reporting. Paired sample *t* tests were used to examine the associations between responses to the in‐person versus the online TLFB for these outlined ancillary measures to further assess the reliability of the O‐TLFB.

Sample size was selected to permit analysis of the primary research questions at two‐tailed *α* of .05 and power level of .80. Prior studies have reported correlations among online and in‐person TLFB assessments that range from *r* = .85–.93 for alcohol and marijuana use variables (Pedersen et al., 2012; Rueger et al., 2012). In addition, mean differences in the range of small to medium effects (*d* = 0.28–0.30) have been found between online TLFB and in‐person TLFB reporting (Pedersen et al., 2012). Based on these effect sizes, we used G*Power a priori to estimate a minimum of 45 individuals completing both the online and in‐person versions would be required to detect a difference in a medium effect size between the two versions. Using a randomization table developed with the use of an online randomization tool (http://researchrandomizer.org), participants were block‐randomized by gender into Groups A (the in‐person TLFB was administered before the online version) and B (the online version was administered before in the in‐person version), in an effort to address order effects. Consistent with the literature concerning multiple comparisons in the context of exploratory studies, we did not adjust our alpha level for multiple comparisons (Althouse, 2016; Feise, 2002; Perneger, 1998; Rothman, 1990). Correcting for multiple comparisons in the context of mean comparisons, in particular, would exacerbate the probability of type II errors, causing us to erroneously determine that there was no mean difference between the two methods.

## 3 RESULTS

**Figure 2:**
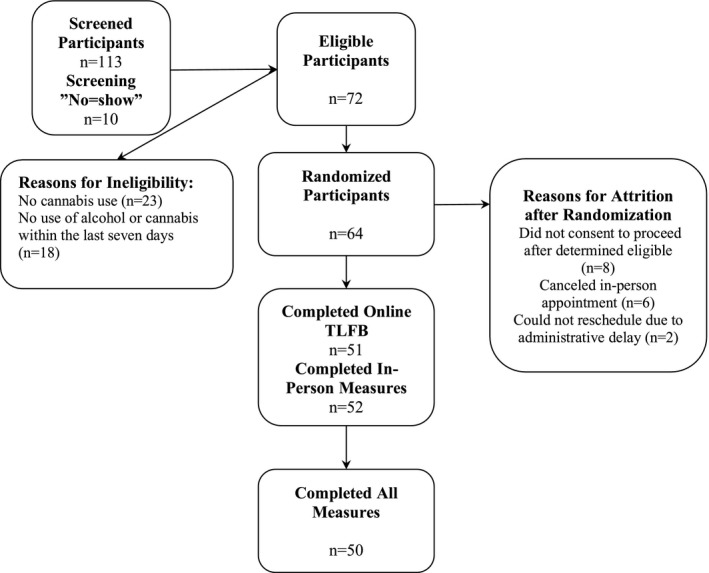
Study recruitment flow

The validation study was completed between February 2018 and June 2018 among undergraduate students in Boulder, CO. One hundred thirteen students participated in an eligibility screening phone call. The final sample consisted of 50 students (*n* = 50; see Figure 2 for study recruitment flow). The demographics of the sample are described in Table 1. Substance use habits among participants were diverse and included alcohol, cannabis, nicotine/tobacco, illicit drug, prescription drugs, and combinations thereof. Means and standard deviations for reported frequency and quantity of substance use over the last 14 days for the online TLFB and in‐person TLFB are displayed in Table 2. Forty‐nine of 50 participants reported use of cannabis, and 48 participants reported use of alcohol. Because the use of nicotine or tobacco was reported relatively infrequently (*n* = 25), the use of any form of nicotine or tobacco (e.g., cigarettes, e‐cigarettes, chew/dip, cigars, and hookah) was collapsed into a single category of nicotine/tobacco use. The same was true of prescription (*n* = 7) and illicit drug use (*n* = 8). There were no significant differences (*p* = .403) in age between the group that completed the in‐person TLFB first (*M* = 19.1 years, *n* = 20) and the group that completed the O‐TLFB first (*M* = 19.4 years, *n* = 28). Two participants declined to disclose their age. Table 2 provides the means and standard deviations as well as the results of the correlation and *t* test analyses comparing the two TLFB modalities for all primary and secondary measures.

**Table 1:** Study demographics

Characteristics	Frequency
*N*	50
Age (*M* (*SD*) years)	19.27 (1.18)
Gender	
Female	20 (40%)
Male	30 (60%)
Race	
White/Caucasian	43 (86.0%)
Asian	5 (10.0%)
Hispanic or Latino	7 (14.0%)
Unknown or not reported	1 (2.0%)
More than one race	1 (2.0%)
Ethnicity	
Hispanic or Latino	7 (14.0%)

**Table 2:** Substance use reported during online TLFB compared with In‐Person TLFB

	Online TLFB		In‐person TLFB		*p*‐value	Correlation
	Mean	*SD*	Mean	*SD*		
Days of cannabis use	7.78	4.71	7.92	4.62	.635	.902**
Days of flower cannabis use	6.46	4.72	6.54	4.72	.813	.873**
Cannabis flower consumed (g)	5.34	6.78	5.79	8.59	.548	.804**
Days of concentrate cannabis use	1.50	3.16	1.68	3.21	.237	.944**
Days of edible cannabis use	0.44	1.07	0.36	0.94	.399	.789**
Days of alcohol use	4.02	2.72	4.24	2.54	.115	.886**
Total drinks	23.38	18.02	28.14	25.74	.023*	.852**
Drinks per drinking day	5.47	2.66	5.94	3.23	.115	.780**
Days of nicotine/tobacco use	3.46	5.12	4.36	5.23	.022*	.864**
Total units of nicotine/tobacco	43.80	72.18	41.98	66.15	.835	.833**
Nicotine/tobacco units per day	3.93	5.22	3.58	4.73	.621	.780**
Days of prescription drug use	0.80	2.81	0.72	2.16	.794	.653**
Days of illegal drug use	0.34	0.77	0.28	0.70	.322	.838**

Note

Pearson correlations reported for all correlations using a 95% confidence interval. *p*‐value calculated from paired sample *t* test between online TLFB and In‐person TLFB substance use reports.

Abbreviation: TLFB, Timeline Followback.

* *p* < .05.

** *p* < .001.

### 3.1 Primary measures

All primary measures, which consisted of days that cannabis, alcohol, nicotine/tobacco, illicit drugs, or prescription drugs were used, were highly correlated between the TLFB modalities (*r *= .902–.653, *p* < .001, *d* = 0.3–0.33). Reported number of days of use using a 95% confidence interval did not differ significantly between TLFB forms (*p* > .05) except for days of nicotine/tobacco use (*t*(49) = 2.359, *p* = .022), where participants reported more days of use in person (*M* = 4.36, *SD* = 5.23) than during the O‐TLFB (*M* = 3.46, *SD* = 5.12).

### 3.2 Secondary measures

All secondary measures, which consisted of days of use across the distinct forms of cannabis (e.g., flower, concentrates, and edibles), as well as the amount of specific forms of cannabis, alcohol, and nicotine/tobacco consumed per occasion, also correlated highly between TLFB modalities (*r* = .944–.780, *p* < .001, *d* = 0.04–0.34). Reported number of days and amounts of substances used largely did not differ significantly between TLFB forms (*p* > .05) except for the total number of alcoholic drinks consumed (*t*(47) = 2.349, *p* = .023) where participants endorsed consuming more in‐person (*M* = 28.14, *SD* = 25.74) than during the O‐TLFB (*M* = 23.38, *SD* = 18.02).

### 3.3 Concurrent validity

The concurrent validity of the O‐TLFB and in‐person TLFB was assessed by examining reported alcohol use in relation to AUDIT scores and reported cannabis use in relation to MDS scores. Those participants who scored above a 2 on the MDS, which signifies the diagnostic cutoff for marijuana dependence (*n* = 31), reported significantly more days of cannabis use on both the in‐person TLFB (*M* = 8.94, *SD* = 4.4, *p* = .046) and the O‐TLFB (*M* = 8.93, *SD* = 4.67, *p* = .025) than participants who did not. Those that were under the diagnostic cutoff for marijuana dependence (*n* = 19) reported on average 6.26 days (*SD* = 4.49) and 5.89 days (*SD* = 4.43) of cannabis use on the in‐person and online TLFBs, respectively. Similarly, participants who scored above an 8 on the AUDIT, which signifies harmful drinking (*n* = 34), reported consuming significantly more total drinks in person (*M* = 32.75, *SD* = 12.18,* p* = .033) and on the O‐TLFB (*M* = 26.73, *SD* = 19.78, *p* = .018) than participants who did not. Participants who scored under an 8 on the AUDIT (*n* = 16) reported on average 18.37 total drinks (*SD* = 16.11) and 16.00 total drinks (*SD* = 10.53) on the in‐person TLFB and O‐TLFB, respectively.

### 3.4 Ancillary findings—cannabis potency and marker date use

#### 3.4.1 Cannabis potency

In states with legal cannabis markets, all products purchased at a cannabis dispensary are required to report THC and CBD potency as determined by a state‐licensed laboratory; thus, users who purchased cannabis at a dispensary should be able to report cannabis potency with reasonable certainty. In regard to cannabis potency, few participants reported a specific potency for their flower. Of those that endorsed flower use during the in‐person TLFB (*n *= 47), 15 participants reported a specific THC potency (*M* = 22.49%, *SD* = 3.85) and three participants reported a specific CBD potency (*M* = 1.40%, *SD* = 0.70). The same numbers reported a specific THC potency (*n* = 15; *M* = 22.61%, *SD* = 3.89) and a specific CBD potency (*n* = 3; *M* = 1.18%, *SD* = 0.31) on the O‐TLFB.

Of those that endorsed concentrated cannabis use during the in‐person TLFB (*n* = 20), seven participants reported a THC potency (*M* = 73.07%, *SD* = 25.47) and three participants reported a CBD potency (*M* = 6.67%, *SD* = 6.65). Similarly, of those that endorsed concentrated cannabis use during the O‐TLFB (*n* = 18), seven participants reported a THC potency (*M* = 67.62%, *SD* = 24.14) and three participants reported a CBD potency (*M* = 5.92%, *SD* = 7.00). The THC and CBD potencies did not differ significantly across the two TLFB forms.

Of those that endorsed edible cannabis use during the in‐person TLFB (*n* = 9), eight participants reported a THC potency (*M* = 19.69 mg, *SD* = 16.71) and two participants reported a CBD potency (*M* = 5.00, *SD* = 7.07). Similarly, of those that endorsed edible cannabis use during the O‐TLFB (*n* = 11), eight participants reported a THC potency (*M* = 21.5 mg, *SD* = 19.63) and two participants reported a CBD potency (*M* = 5.00, *SD* = 7.07).

#### 3.4.2 Marker dates

While it was clearly stated in the beginning of both the online and in‐person assessments that marker dates should be reported, participants utilized them on the O‐TLFB significantly more (*p* = .016) than during the in‐person TLFB. On average, participants used 1.12 (*SD* = 1.21) marker dates during the in‐person TLFB and 2.80 (*SD* = 4.46) during the online TLFB. Furthermore, there were group differences in the use of marker dates. Those who completed the O‐TLFB prior to the in‐person TLFB used significantly more marker dates (*p* = .004) on the O‐TLFB (*M* = 4.95) compared with the in‐person TLFB (*M* = 0.956). Those who completed the in‐person TLFB first used a similar number of marker dates (*p* = .396) on the O‐TLFB (*M* = 1.25) and the in‐person TLFB (*M* = 1.11).

#### 3.4.3 Order effects

When examining order effects, there were largely no differences between those who were randomized to complete the O‐TLFB first and those who were randomized to complete the in‐person TLFB first, with two exceptions. On the in‐person TLFB, participants in the group that completed the in‐person version first reported significantly more (*p* = .035) Cannabis use days (*M* = 9.52, *SD* = 4.72) than those participants who completed the in‐person TLFB second (*M* = 6.76, *SD* = 4.26). With regard to marker dates, the group that completed the in‐person TLFB first used significantly more marker dates (*p* = .004) on the O‐TLFB (*M* = 4.95) compared with the in‐person TLFB (*M *= 0.956). Participants who completed the online version first used a similar number of marker dates (*p* = .396) on the O‐TLFB (*M* = 1.25) and the in‐person TLFB (*M *= 1.11).

## 4 DISCUSSION

The TLFB continues to be a useful tool for assessing substance use quantity and frequency patterns, within the broader scope of validated substance use assessments that each vary on their research‐based and clinical utility (Carey, 2002). Importantly, the TLFB is not a diagnostic tool, but reveals frequency and quantity patterns and has the potential to measure fine‐grained details of use, especially for substances that are newly emerging (such as e‐cigarettes or edible cannabis products for instance) and for which the field is still exploring the best way to examine and quantify.

While popular and well‐validated, the TLFB requires a relatively labor‐intensive in‐person administration. Further, while situations remain where an in‐person administration of this assessment is more appropriate than an online or computer‐based version (e.g., among patient groups or with respondents who have low computer literacy), an online assessment has many advantages. It may be more appropriate for a large community sample, or for multiple administrations across many months or years, and it increases participant convenience and privacy. The online format is easy and intuitive to use with basic Internet literacy possessed by nearly all Americans (Ryan, 2016), and finally, it offers uniformity of data capture and seamlessly integrates with REDCap, a method of data management that is increasingly utilized in biological and behavioral research, including clinical settings (Bahr, Christensen, Agarwal, George, & Bhutani, 2019; Blough, Mansfield, & Kondapalli, 2014; Campion, Sholle, & Davila, 2017; Cantor, Plint, Kamil, & Zemek, 2016; Dunn et al., 2016; Gabriel, Finlay, & Weiner, 2012; Obeid et al., 2013; Yamamoto et al., 2017). Data obtained from the O‐TLFB demonstrated a high level of consistency with the in‐person TLFB interview and strong concurrent validity with the MDS and AUDIT. Our findings extend previous research on the feasibility and validity of administering the TLFB in an online format (Hjorthøj, Fohlmann, Larsen, Arendt, & Nordentoft, 2012; Hoeppner et al., 2010; Rueger et al., 2012).

Of utmost importance, this O‐TLFB has the ability to query an increased diversity of substances (e.g., prescription drugs and illicit drugs) in greater detail in terms of method of administration (e.g., cannabis could be endorsed through flower, concentrate, or edible use and nicotine/tobacco could be endorsed through cigarettes, e‐cigarettes, chew or dip, cigars, hookah, or other tobacco), dosage (differentiated according to mode of administration, e.g., pinch, puff, and drag), and potency (e.g., THC vs. CBD proportions in cannabis products), and in flexible combinations based on the need of the study (i.e., one study may query prescription medications while another might omit them, and one study may query 14 retrospective days awhile another may query 60). Capturing these specifics online as accurately as the in‐person version of the TLFB is important in today's climate of increased legalization and accessibility of cannabis, use of prescription medications recreationally, and rapidly changing forms of nicotine consumption. It is interesting to note, however, that in the course of the validation of the O‐TLFB, an ancillary finding regarding marker date use (a retrospective calendar as a memory “trigger” for instances of substance use) became apparent. While it was shown that participants made greater use of marker dates online than in person, overall the results indicated that the two modalities are highly correlated across primary and secondary substance use measures despite this difference.

Our study should be interpreted in the context of the following limitations. First, disclosures of the true intent to compare the TLFB methods were given to participants at the end of their in‐person interview. Therefore, participants who interviewed before taking the O‐TLFB had the chance to read the disclosure and understand the purpose of the study before they completed the O‐TLFB, potentially influencing the study's results. Despite this, however, substance use reporting levels across both groups were consistently related across all domains.

Second, the location of the study in Colorado—a state that has legalized the medical and recreational use of cannabis—and the use of a convenience sample of college students are also limitations that may have also influenced results. For example, while we demonstrated preliminary reliability for the reporting of both quantity and frequency across multiple forms of substances, the differences between the online and in‐person measures for nicotine/tobacco total days and alcohol total drinks were significantly different. This could be due to the fact that use in our sample was notably limited, though it should be noted that the actual differences were small and do not appear to be clinically meaningful and could benefit from further validation among community or clinical samples. Also, while our measure demonstrated reliable reporting of days and amounts used for different modes of cannabis administration, such as flower, edibles, and concentrates, the majority of participants interestingly did not report on the specific THC or CBD contents of their cannabis as demonstrated by our ancillary findings. This limitation could be due to our reliance on a sample that was on average below the legal age for purchasing and using cannabis (21), and thus more likely to use cannabis in groups socially where potency is not known by users. Therefore, these potency measures could be more powerful in studies of older or medical cannabis users or heavier substance users. Again, this validation study using a convenience sample is a first step toward demonstrating the validity of these novel potency and administration measures among diverse samples.

Finally, participants may also have been more comfortable and had more knowledge reporting on their cannabis and other substance use in our state, as a result of the state cannabis packaging regulations and general social climate surrounding use, which in turn may limit the replicability of this study in other states. Future directions would include additional validation with a community or clinical sample to further explore the validity of substance categories measured by the O‐TLFB. Overall, however, the data suggest that this new O‐TLFB demonstrates reliability across a wide variety of substances in greater detail. It delivers a useful addition to the broader substance use measurement arsenal that is relevant to a variety of biomedical and psychological research contexts.

## CONFLICT OF INTEREST

The authors declare that they have no conflict of interest.

## Data Availability

The data that support the findings of this study are available from the corresponding author upon reasonable request.
