# Supplementary material for: Validation of a multisubstance online Timeline Followback assessment
Source: Brain Behav. 2019 Dec 2;10(1):e01486. doi: 10.1002/brb3.1486 (PMC6955818; doi:10.1002/brb3.1486)
Supplement: Supplementary file 1 [file BRB3-10-e01486-s001.docx]

**Online TIMELINE FOLLOW-BACK**

LOG-IN/INTRODUCTION

*(TLFB Sign-In page)*

***PROMPT:*** Please enter the last five digits of your phone number and then the first letter of your first name to access the Timeline Follow Back Survey.

*XXXXX Last 5 digits*

*XXXXX Confirm last 5 digits*

*X First Letter of your first name*

*X Confirm first Letter of your first name*

- CLICK ON SIGN IN TO BEGIN

*(TLFB Landing Page Blurb/Consent)*

***PROMPT:*** "For this exercise, you will be asked about your use of various substances (e.g., alcohol, cannabis, recreational drugs, etc.) over the past 14 days. You will be asked about medical and recreational use of cannabis, as well as any substances you take recreationally. You will be entering responses into a calendar that is programmed to show you the last 14 days." The page you are on now is to help you remember what you did on a certain day. [You will not enter the substances you have used until the next page.] To complete this page, please also use personal items such as text messages or your personal calendar to help you remember. You can mark any memorable events that occurred (e.g. birthdays, holidays, parties, concert, camping trip) by clicking the box that says “**Date of important or memorable event in the last 2 weeks**” and choosing the date it occurred from the calendar that will appear, and then clicking the box that says “**Event name**” and naming the event (e.g., “Birthday” or “Red Rocks concert.”) Then click **"Add Marker."** This event will now show up on the next page as well as this page.

Once you click “Next page” below, you will go to the page where you enter the substances you’ve used. For each day on the calendar you will click **"Enter Substances"** and check the boxes next to the listed substances to indicate all substances you used that day. Then, you will be prompted to answer more detailed questions related to each item that you selected (e.g., What type of tobacco did you use? How many cigarettes did you smoke?). When you can't remember certain days or details perfectly, it is okay. For example, if you had about 8-10 drinks last Thursday or Friday but can't be sure, just give it your best guess. What is important is that 8 or 10 drinks is very different than 1 or 20 drinks.

“Once you have added markers and entered substances for a particular day, please press **"Submit"** for that day. Note, if you made a mistake on any day on the calendar, you can press "Re-Enter Substances" to re-do all of your responses for that day. Warning: Pressing **"Re-enter Substances"** will erase all of your responses for that particular day and you will be asked to Re-enter your substance use for that day. After you have completed all days to the best of your knowledge, please press **"Submit all responses"**. The survey should take you about 20 minutes. **Please, try to be as accurate as possible.** Thank you for your help!

CLICK ON NEXT PAGE TO MOVE ON FROM MARKER DAYS TO SUBSTANCE ENTRY

*(Complete marker days with calendar in view)*

***PROMPT: After marking events and recording your answers for each day, you will see a green check mark in each day, and the grayed out “Submit All Responses” button below the calendar will become available to click.***

***Please press “Submit All Responses” when you are ready to submit all of your answers.***

- CLICK ON DAY 1 TO BEGIN

DROP-DOWNS/POP-UPS FOR: SUBSTANCES

*(Day 1)*

***PROMPT: Were any substances used on this day? If so, please select all of the substances used.***

***If no, please select None.***

- **Alcohol** (cannabis infused alcohol, beer, wine, hard liquor, etc.)
- **Tobacco** (cigarettes, e-cigarettes, chew/dip, cigars, hookah, dokha, nicotine patch, etc.)
- **Cannabis** (include medical AND recreational use of flower/bud, edibles, concentrates/dabs, topicals, etc.)
- **Illegal recreational drugs** (i.e. cocaine, LSD, etc.)
- **Other Substances** (recreational use of prescription drugs, salvia, kratom, synthetic cannabis (K2, spice), etc.)
- **None** (I did not use any substances on this day)
- CLICK ON SUBMIT FOR THIS 1 LARGE CATEGORY WHICH BRANCHES TO BELOW CATEGORIES (AS APPLICABLE, SEE COMMENT TO THE RIGHT TO SEE FLOW/ORDER)

DROP-DOWNS/POP-UPS FOR: TYPES

*(Day1>Alcohol)*

***PROMPT: What type of alcohol did you have?***

- **Beer**
- **Wine/Champagne**
- **Hard liquor** (or mixed drinks)
- **Other/Alcohol, please fill in type** (include liqueur (e.g. schnapps), cannabis infused beer, cannabis infused hard liquor, etc.): ______

*(Day 1>Tobacco)*

***PROMPT: What type of tobacco did you use?***

- **Cigarettes**
- **E-Cigs**
- **Chew/dip**
- **Cigar**
- **Hookah**
- **Other/Tobacco (include dokha, nicotine patch, etc.): ______**

*(Day 1>Cannabis (Study Product))*

***PROMPT: What type of Study cannabis product did you use?***

- **Cannabis flower/bud** (include using a pipe, bong, bubbler, joint, blunt, spliff, vaporizer, etc.)
- **Cannabis edible/ingested** (include eating a piece of chocolate bar, gummy, baked good, capsule, tincture, etc.)

*(Day 1>Cannabis (Non-Study Product))*

***PROMPT: What type of Non-Study cannabis product did you use?***

- **Cannabis flower/bud** (include using a pipe, bong, bubbler, joint, blunt, spliff, vaporizer)
- **Cannabis ingested/edible** (include eating a piece of chocolate bar, gummy, baked good, capsule, tincture)
- **Cannabis concentrate** (include shatter, wax, vape pen cartridges, disposable vape pen, etc.)
- **Other Non-study Cannabis** (include topical creams, topical body oils, transdermal patches, bath bombs, vaginal suppositories, personal lubricant, etc.)
  - **Non-study Cannabis - Other**
    - **Non-study cannabis topical/patch** (include topical creams, topical body oils, transdermal patches, bath bombs, etc.)
    - **Other non-study cannabis name** (include vaginal suppositories, personal lubricant, etc.)

*(Day 1>Illegal drugs)*

***PROMPT: What illegal drugs did you use?***

- **Cocaine**
- **Amphetamine**
- **Methamphetamine**
- **MDMA**
- **Heroin**
- **LSD**
- **Mushrooms**
- **Peyote**
- **Ecstasy**
- **Other/Illegal drugs, please fill in type: _______**

*(Day 1>Other Substances)*

***PROMPT: What other drugs did you use?***

- **Other/Substances:** (Please fill in type and amount. Include any recreational use of prescription drugs, salvia, kratom, synthetic cannabis (K2, spice), etc.)

DROP-DOWNS/POP-UPS FOR: AMOUNTS/POTENCY

*(Day 1>Alcohol>Beer)*

***PROMPT: How many beers did you drink?*** *(consider 1 beer = 12 oz. and 1 US pint = 1 ½ beers)*

DROP-DOWN OPTIONS:

- ≤½ beer
- 1 beer
- 1 ½ beers
- 2 beers
- 2 ½ beers
- 3 beers
- 3 ½ beers
- 4 beers
- 4 ½ beers
- 5 beers
- 6 beers
- 7 beers
- 8 beers
- 9 beers
- 10 beers
- 11 beers
- 12 beers
- 13 beers
- 14 beers
- 15 beers
- 16 beers
- 17 beers
- 18 beers
- 19 beers
- 20 or more beers

*(Day1>Alcohol>Wine)*

***PROMPT: How many glasses of wine did you drink?*** *(consider 1 glass of wine = 5 oz.)*

DROP-DOWN OPTIONS:

- ≤½ glass of wine
- 1 glass of wine
- 1 ½ glasses of wine
- 2 glasses of wine
- 2 ½ glasses of wine
- 3 glasses of wine
- 3 ½ glasses of wine
- 4 glasses of wine
- 4 ½ glasses of wine
- 5 glasses of wine
- 6 glasses of wine
- 7 glasses of wine
- 8 glasses of wine
- 9 glasses of wine
- 10 glasses of wine
- 11 glasses of wine
- 12 glasses of wine
- 13 glasses of wine
- 14 glasses of wine
- 15 glasses of wine
- 16 glasses of wine
- 17 glasses of wine
- 18 glasses of wine
- 19 glasses of wine
- 20 or more glasses of wine

*(Day1>Alcohol>Hard Liquor)*

***PROMPT: How many shots (or mixed drinks) of hard liquor did you have?*** *(consider 1 shot = 0.5 oz., drinks are usually more than 1 shot)*

DROP-DOWN OPTIONS:

- ≤½ shot
- 1 shot
- 1 ½ shots
- 2 shots
- 2 ½ shots
- 3 shots
- 3 ½ shots
- 4 shots
- 4 ½ shots
- 5 shots
- 6 shots
- 7 shots
- 8 shots
- 9 shots
- 10 shots
- 11 shots
- 12 shots
- 13 shots
- 14 shots
- 15 shots
- 16 shots
- 17 shots
- 18 shots
- 19 shots
- 20 or more shots

*(*Day 1>Alcohol>*Other/Alcohol)*

***PROMPT: Other/Alcohol, please fill in the potency, content, or amount of what you used with units*** *(i.e., drinks, mg, grams, ml, %, etc.): _____*

*(Day 1>Tobacco>Cigarettes)*

***PROMPT: How many cigarettes did you smoke?*** *(consider 20 cigs = 1 pack)*

DROP-DOWN OPTIONS:

- - ≤½ cigarette
  - 1 cigarette
  - 1 ½ cigarettes
  - 2 cigarettes
  - 2 ½ cigarettes
  - 3 cigarettes
  - 3 ½ cigarettes
  - 4 cigarettes
  - 4 ½ cigarettes
  - 5 cigarettes
  - 6 cigarettes
  - 7 cigarettes
  - 8 cigarettes
  - 9 cigarettes
  - 10 cigarettes
  - 11 cigarettes
  - 12 cigarettes
  - 13 cigarettes
  - 14 cigarettes
  - 15 cigarettes
  - 16 cigarettes
  - 17 cigarettes
  - 18 cigarettes
  - 19 cigarettes
  - 20 or more cigarettes

(Day 1>Tobacco>*E-Cigs*)

***PROMPT: How many times did you use your e-cigarette?*** *(consider 1 e-cigarette use= ~10 minutes of smoking or 15 puffs)*

DROP-DOWN OPTIONS:

- - ≤¼ e-cigarette
  - ½ e-cigarette
  - ¾ e-cigarette
  - 1 e-cigarette
  - 1 ½ e-cigarettes
  - 2 e-cigarettes
  - 2 ½ e-cigarettes
  - 3 e-cigarettes
  - 3 ½ e-cigarettes
  - 4 e-cigarettes
  - 4 ½ e-cigarettes
  - 5 e-cigarettes
  - 6 e-cigarettes
  - 7 e-cigarettes
  - 8 e-cigarettes
  - 9 e-cigarettes
  - 10 e-cigarettes
  - 11 e-cigarettes
  - 12 e-cigarettes
  - 13 e-cigarettes
  - 14 e-cigarettes
  - 15 e-cigarettes
  - 16 e-cigarettes
  - 17 e-cigarettes
  - 18 e-cigarettes
  - 19 e-cigarettes
  - 20 or more e-cigarettes

*(*Day 1>Tobacco>*Chew)*

***PROMPT: How many times did you chew?*** *(consider number of pinches or dips)*

DROP-DOWN OPTIONS:

- - ≤½ pinch/dip
  - 1 pinch/dip
  - 1 ½ pinches/dips
  - 2 pinches/dips
  - 2 ½ pinches/dips
  - 3 pinches/dips
  - 3 ½ pinches/dips
  - 4 pinches/dips
  - 4 ½ pinches/dips
  - 5 pinches/dips
  - 6 pinches/dips
  - 7 pinches/dips
  - 8 pinches/dips
  - 9 pinches/dips
  - 10 pinches/dips
  - 11 pinches/dips
  - 12 pinches/dips
  - 13 pinches/dips
  - 14 pinches/dips
  - 15 pinches/dips
  - 16 pinches/dips
  - 17 pinches/dips
  - 18 pinches/dips
  - 19 pinches/dips
  - 20 or more pinches/dips

*(*Day 1>Tobacco>*Cigars)*

***PROMPT: How many cigars did you smoke?***

DROP-DOWN OPTIONS:

- - ≤¼ cigar
  - ½ cigar
  - ¾ cigar
  - 1 cigar
  - 1 ½ cigars
  - 2 cigars
  - 2 ½ cigars
  - 3 cigars
  - 3 ½ cigars
  - 4 cigars
  - 4 ½ cigars
  - 5 cigars
  - 6 cigars
  - 7 cigars
  - 8 cigars
  - 9 cigars
  - 10 or more cigars

*(*Day 1>Tobacco>*Hookah)*

***PROMPT: How many hookah drags/puffs did you have?***

DROP-DOWN OPTIONS:

- - ≤½ drag/puff
  - 1 drag/puff
  - 1 ½ drags/puffs
  - 2 drags/puffs
  - 2 ½ drags/puffs
  - 3 drags/puffs
  - 3 ½ drags/puffs
  - 4 drags/puffs
  - 4 ½ drags/puffs
  - 5 drags/puffs
  - 6 drags/puffs
  - 7 drags/puffs
  - 8 drags/puffs
  - 9 drags/puffs
  - 10 drags/puffs
  - 11 drags/puffs
  - 12 drags/puffs
  - 13 drags/puffs
  - 14 drags/puffs
  - 15 drags/puffs
  - 16 drags/puffs
  - 17 drags/puffs
  - 18 drags/puffs
  - 19 drags/puffs
  - 20 drags/puffs
  - 30 drags/puffs
  - 40 drags/puffs
  - 50 drags/puffs
  - 60 drags/puffs
  - 70 drags/puffs
  - 80 or more drags/puffs

*(*Day 1>Tobacco>*Other/Tobacco)*

***PROMPT: Other/Tobacco, (include dokha, nicotine patch, etc.) please fill in the potency, content, or amount of what you used with units*** *(i.e., mg, grams, ml, %, etc.): _____*

*(Day 1>Cannabis Study Product>Flower)*

***PROMPT: How many total grams did you use (e.g., smoked)?***

DROP-DOWN OPTIONS:

- - ≤0.05 grams
  - 0.1 grams
  - 0.15 grams
  - 0.20 grams
  - 0.25 grams (1/4/ gram)
  - 0.30 grams
  - 0.35 grams
  - 0.40 grams
  - 0.45 grams
  - 0.50 grams (1/2/ gram)
  - 0.55 grams
  - 0.60 grams
  - 0.65 grams
  - 0.70 grams
  - 0.75 grams (3/4 gram)
  - 0.80 grams
  - 0.85 grams
  - 0.90 grams
  - 0.95 grams
  - 1 gram
  - 1.1 grams
  - 1.2 grams
  - 1.3 grams
  - 1.4 grams
  - 1.5 grams
  - 1.6 grams
  - 1.7 grams
  - 1.8 grams
  - 1.9 grams
  - 2 grams
  - 2.1 grams
  - 2.2 grams
  - 2.3 grams
  - 2.4 grams
  - 2.5 grams
  - 2.6 grams
  - 2.7 grams
  - 2.8 grams
  - 2.9 grams
  - 3 grams
  - 3.0 gram
  - 3.1 grams
  - 3.2 grams
  - 3.3 grams
  - 3.4 grams
  - 3.5 grams
  - 3.6 grams
  - 3.7 grams
  - 3.8 grams
  - 3.9 grams
  - 4 grams
  - 4.1 grams
  - 4.2 grams
  - 4.3 grams
  - 4.4 grams
  - 4.5 grams
  - 4.6 grams
  - 4.7 grams
  - 4.8 grams
  - 4.9 grams
  - 5 grams
  - 6 grams
  - 7 grams
  - 8 grams
  - 9 grams
  - 10 grams
  - 11 grams
  - 12 grams
  - 13 grams
  - 14 grams
  - 15 grams
  - 16 grams
  - 17 grams
  - 18 grams
  - 19 grams
  - 20 or more grams of flower

*(Day 1>Cannabis Study Product>Edible)*

***PROMPT: How many total THC milligrams (best guess) did you use consume (e.g., eat)?:***

DROP-DOWN OPTIONS:

- - Unknown
  - ≤1 mg THC
  - 1 ½ mg THC
  - 2 mg THC
  - 2 ½ mg THC
  - 3 mg THC
  - 3 ½ mg THC
  - 4 mg THC
  - 4 ½ mg THC
  - 5 mg THC
  - 6 mg THC
  - 7 mg THC
  - 8 mg THC
  - 9 mg THC
  - 10 mg THC
  - 15 mg THC
  - 20 mg THC
  - 25 mg THC
  - 30 mg THC
  - 35 mg THC
  - 40 mg THC
  - 45 mg THC
  - 50 mg THC
  - 60 mg THC
  - 70 mg THC
  - 80 mg THC
  - 90 mg THC
  - 100 mg THC
  - 125 mg THC
  - 150 mg THC
  - 175 mg THC
  - 200 or more mg THC

***PROMPT: How many total CBD milligrams (best guess) did you use consume (e.g., eat)?:***

DROP-DOWN OPTIONS:

- - Unknown
  - ≤1 mg CBD
  - 1 ½ mg CBD
  - 2 mg CBD
  - 2 ½ mg CBD
  - 3 mg CBD
  - 3 ½ mg CBD
  - 4 mg CBD
  - 4 ½ mg CBD
  - 5 mg CBD
  - 6 mg CBD
  - 7 mg CBD
  - 8 mg CBD
  - 9 mg CBD
  - 10 mg CBD
  - 15 mg CBD
  - 20 mg CBD
  - 25 mg CBD
  - 30 mg CBD
  - 35 mg CBD
  - 40 mg CBD
  - 45 mg CBD
  - 50 mg CBD
  - 60 mg CBD
  - 70 mg CBD
  - 80 mg CBD
  - 90 mg CBD
  - 100 mg CBD
  - 125 mg CBD
  - 150 mg CBD
  - 175 mg CBD
  - 200 or more mg CBD

*(Day 1>Cannabis Non-Study Product>Flower)*

***PROMPT: How many total grams did you use (e.g., smoked)?***

DROP-DOWN OPTIONS:

- - ≤0.05 grams
  - 0.1 grams
  - 0.15 grams
  - 0.20 grams
  - 0.25 grams (1/4/ gram)
  - 0.30 grams
  - 0.35 grams
  - 0.40 grams
  - 0.45 grams
  - 0.50 grams (1/2/ gram)
  - 0.55 grams
  - 0.60 grams
  - 0.65 grams
  - 0.70 grams
  - 0.75 grams (3/4 gram)
  - 0.80 grams
  - 0.85 grams
  - 0.90 grams
  - 0.95 grams
  - 1 gram
  - 1.1 grams
  - 1.2 grams
  - 1.3 grams
  - 1.4 grams
  - 1.5 grams
  - 1.6 grams
  - 1.7 grams
  - 1.8 grams
  - 1.9 grams
  - 2 grams
  - 2.1 grams
  - 2.2 grams
  - 2.3 grams
  - 2.4 grams
  - 2.5 grams
  - 2.6 grams
  - 2.7 grams
  - 2.8 grams
  - 2.9 grams
  - 3 grams
  - 3.0 gram
  - 3.1 grams
  - 3.2 grams
  - 3.3 grams
  - 3.4 grams
  - 3.5 grams
  - 3.6 grams
  - 3.7 grams
  - 3.8 grams
  - 3.9 grams
  - 4 grams
  - 4.1 grams
  - 4.2 grams
  - 4.3 grams
  - 4.4 grams
  - 4.5 grams
  - 4.6 grams
  - 4.7 grams
  - 4.8 grams
  - 4.9 grams
  - 5 grams
  - 6 grams
  - 7 grams
  - 8 grams
  - 9 grams
  - 10 grams
  - 11 grams
  - 12 grams
  - 13 grams
  - 14 grams
  - 15 grams
  - 16 grams
  - 17 grams
  - 18 grams
  - 19 grams
  - 20 or more grams flower

***PROMPT: What was the flower THC potency (best guess, %, 0-35): ____***

- - Unknown

≤1% THC

2% THC

3% THC

4% THC

5% THC

6% THC

7% THC

8% THC

9% THC

10% THC

11% THC

12% THC

13% THC

14% THC

15% THC

16% THC

17% THC

18% THC

19% THC

20% THC

21% THC

22% THC

23% THC

24% THC

25% THC

26% THC

27% THC

28% THC

29% THC

30% THC

31% THC

32% THC

33% THC

34% THC

35% or more THC

***PROMPT: What was the flower CBD potency (best guess, %, 0-35): ____***

- - Unknown

≤1% CBD

2% CBD

3% CBD

4% CBD

5% CBD

6% CBD

7% CBD

8% CBD

9% CBD

10% CBD

11% CBD

12% CBD

13% CBD

14% CBD

15% CBD

16% CBD

17% CBD

18% CBD

19% CBD

20% CBD

21% CBD

22% CBD

23% CBD

24% CBD

25% CBD

26% CBD

27% CBD

28% CBD

29% CBD

30% CBD

31% CBD

32% CBD

33% CBD

34% CBD

35% or more CBD

*(Day 1> Cannabis Non-Study Product>Edible)*

***PROMPT: How many total THC milligrams (best guess) did you use consume (e.g., eat)?:***

DROP-DOWN OPTIONS:

- - Unknown
  - ≤1 mg THC
  - 1 ½ mg THC
  - 2 mg THC
  - 2 ½ mg THC
  - 3 mg THC
  - 3 ½ mg THC
  - 4 mg THC
  - 4 ½ mg THC
  - 5 mg THC
  - 6 mg THC
  - 7 mg THC
  - 8 mg THC
  - 9 mg THC
  - 10 mg THC
  - 15 mg THC
  - 20 mg THC
  - 25 mg THC
  - 30 mg THC
  - 35 mg THC
  - 40 mg THC
  - 45 mg THC
  - 50 mg THC
  - 60 mg THC
  - 70 mg THC
  - 80 mg THC
  - 90 mg THC
  - 100 mg THC
  - 125 mg THC
  - 150 mg THC
  - 175 mg THC
  - 200 or more mg THC

***PROMPT: How many total CBD milligrams (best guess) did you use consume (e.g., eat)?:***

DROP-DOWN OPTIONS:

- - Unknown
  - ≤1 mg CBD
  - 1 ½ mg CBD
  - 2 mg CBD
  - 2 ½ mg CBD
  - 3 mg CBD
  - 3 ½ mg CBD
  - 4 mg CBD
  - 4 ½ mg CBD
  - 5 mg CBD
  - 6 mg CBD
  - 7 mg CBD
  - 8 mg CBD
  - 9 mg CBD
  - 10 mg CBD
  - 15 mg CBD
  - 20 mg CBD
  - 25 mg CBD
  - 30 mg CBD
  - 35 mg CBD
  - 40 mg CBD
  - 45 mg CBD
  - 50 mg CBD
  - 60 mg CBD
  - 70 mg CBD
  - 80 mg CBD
  - 90 mg CBD
  - 125 mg CBD
  - 150 mg CBD
  - 175 mg CBD
  - 200 or more mg CBD

*(Day 1>Cannabis Non-Study Product>Concentrates)*

***PROMPT: How many dabs/drags/hits did you do?***

- DROP-DOWN OPTIONS:
- ≤1 dab/drag/hit
- 2 dabs/drags/hits
- 3 dabs/drags/hits
- 4 dabs/drags/hits
- 5 dabs/drags/hits
- 6 dabs/drags/hits
- 7 dabs/drags/hits
- 8 dabs/drags/hits
- 9 dabs/drags/hits
- 10 dabs/drags/hits
- 11 dabs/drags/hits
- 12 dabs/drags/hits
- 13 dabs/drags/hits
- 14 dabs/drags/hits
- 15 dabs/drags/hits
- 16 dabs/drags/hits
- 17 dabs/drags/hits
- 18 dabs/drags/hits
- 19 dabs/drags/hits
- 20 dabs/drags/hits
- 25 dabs/drags/hits
- 30 dabs/drags/hits
- 35 dabs/drags/hits
- 40 or more dabs/drags/hits

***PROMPT: What was the concentrate THC potency (best guess, %, 0-100)?: _____***

- - Unknown

≤1% THC

2% THC

3% THC

4% THC

5% THC

6% THC

7% THC

8% THC

9% THC

10% THC

11% THC

12% THC

13% THC

14% THC

15% THC

16% THC

17% THC

18% THC

19% THC

20% THC

21% THC

22% THC

23% THC

24% THC

25% THC

26% THC

27% THC

28% THC

29% THC

30% THC

31% THC

32% THC

33% THC

34% THC

35% THC

40% THC

45% THC

50% THC

55% THC

60% THC

65% THC

70% THC

75% THC

80% THC

85% THC

90% THC

95% or more THC

***PROMPT: What was the concentrate CBD potency (best guess, %, 0-100)?: ____***

- - Unknown

≤1% CBD

2% CBD

3% CBD

4% CBD

5% CBD

6% CBD

7% CBD

8% CBD

9% CBD

10% CBD

11% CBD

12% CBD

13% CBD

14% CBD

15% CBD

16% CBD

17% CBD

18% CBD

19% CBD

20% CBD

21% CBD

22% CBD

23% CBD

24% CBD

25% CBD

26% CBD

27% CBD

28% CBD

29% CBD

30% CBD

31% CBD

32% CBD

33% CBD

34% CBD

35% CBD

40% CBD

45% CBD

50% CBD

55% CBD

60% CBD

65% CBD

70% CBD

75% CBD

80% CBD

85% CBD

90% CBD

95% or more CBD

VERIFICATION/SUBMISSION

(<<*green check mark in each day now, and the grayed-out REVIEW button below the calendar becomes available to click>>)*

- CLICK ON REVIEW

***Please click the RE-ENTER SUBSTANCES to change your answers and remember to use any other calendar or device to verify your answers.***

(<<*Once all days are completed, a grayed out SUBMIT ALL RESPONSES button becomes available to click>>)*

- CLICK ON SUBMIT ALL RESPONSES

***PROMPT: Thank you so much! We greatly appreciate your time and help completing this!***
